# Loss of nonclassical MHC molecules MIC-A/B expression during progression of uveal melanoma

**DOI:** 10.1038/sj.bjc.6602123

**Published:** 2004-09-21

**Authors:** C S Vetter, W Lieb, E-B Bröcker, J C Becker

**Affiliations:** 1 Department of Dermatology, University of Wuerzburg, Josef-Schneider-Str. 2, 97080 Wuerzburg, Germany; 2 Department of Ophthalmology, St Vincentius-Hospital gAG, Steinhaeußerstr. 18, 76135 Karlsruhe, Germany

**Keywords:** uvea, MIC-A/B, melanoma, NKG2D, NK

## Abstract

Uveal melanoma differs from cutaneous melanoma with respect to aetiology, metastatic behaviour and immune biology. The notion that loss of classical MHC class I molecules in uveal melanoma lesions is associated with an improved prognosis suggests that NK cells act as the predominant cells responsible for immune surveillance of this tumour. Consequently, immune escape mechanisms of uveal melanoma should impair the innate immunity. To this end, expression of the ligand for the NK receptor NKG2D, that is, MIC-A/B was expressed by 50% of primary tumours, but none of the metastatic lesions. MIC^+^ tumours were characterised by a NKG2D^+^ infiltrate, which was absent in MIC^−^ lesions subsequent to chemoimmune therapy. Strikingly, MIC-A/B expression in metastatic lesions was observed subsequent to chemotherapy with fotemustine in one case. In summary, MIC/NKG2D interactions seem to be involved in the immune surveillance of primary uveal melanomas, whereas for metastatic tumours this ligand/receptor system seems not to be relevant, thus, suggesting an immune selection of MIC negative tumour cells.

Most malignant melanomas in the ocular region arise in the uveal tract. Little is known about its underlying molecular pathogenesis since no genes and tumour-suppressor pathways have so far been convincingly linked to it (Edmunds *et al*, 2002). The annual incidence of uveal melanoma is in the range of 0.47–0.79 new cases per 100 000 individuals (Woll *et al*, 1999). Despite its lower incidence compared to cutaneous melanoma, uveal melanoma accounts for about 13% of all deaths from melanoma (Albert *et al*, 1992). Uveal melanoma preferentially disseminates haematogenously to the liver and occurrence of metastases is associated with a median survival time of less than 5 months (Eskelin *et al*, 1999). For cutaneous melanoma substantial advances in the understanding of its pathogenesis have been achieved (Chin *et al*, 1998). Among these is the recent detection of mutations in the BRAF gene (Davies *et al*, 2002). Notably, BRAF is not mutated in uveal melanoma (Edmunds and Kelsell, 2002; Cohen *et al*, 2003), which highlights the molecular differences between uveal and cutaneous melanoma and may explain both the different metastatic behaviour as well as immune biology of these tumours (Naus *et al*, 2000; Soufir *et al*, 2000). This could, for example, be the consequence of reduced HLA class I antigen expression; a decrease thereof in cutaneous melanoma is characteristic for tumour progression and represents an important tumour escape mechanism (Garrido *et al*, 1993; Hicklin *et al*, 1999). Although almost all possible HLA class I antigen loss described for cutaneous melanoma (Algarra *et al*, 2000) may occur in uveal melanomas (Jager *et al*, 2002), it is important to note that metastases of uveal melanoma have a similar or even an increased expression of MHC molecules as compared to the primary tumour (Blom *et al*, 1997b). Moreover, loss of HLA class-I expression in primary tumours is associated with an improved survival and a lower occurrence of metastases (Blom *et al*, 1997a; Ksander and Chen, 1999; Ericsson *et al*, 2001). Since loss of HLA class I antigens renders cells more sensitive to NK cell mediated lysis, it has been speculated that the immune surveillance of uveal melanomas is based on NK cells (Rothenfusser *et al*, 2002).

Over the past years it has been established that activating and inhibiting MHC-specific receptors regulate the activity of NK cells. In this respect, NKG2D differs from most other receptors as it exists only in an activating form. The activating killer receptor NKG2D is expressed on both *α*/*β* and *γ*/*δ* T-cells as well as NK cells (Groh *et al*, 2001). The MHC class I related chain (MIC)-A/B molecules were recently identified as ligands for this receptor. The MIC molecules possess a low degree of homology to other MHC class-I encoded genes (Bahram, 2000). Unlike classical class I molecules MIC-A/B are not associated with *β*_2_-microglobulin and do not bind peptide. MIC-A/B is highly polymorphic in its transmembrane and extracellular region (Stephens, 2001; Collins *et al*, 2002), and only some MIC-A/B variants are indeed expressed on the cell surface. Our recent observation that primary cutaneous melanoma expresses MIC-A/B and that this expression is downregulated in metastatic lesions (Vetter *et al*, 2002) prompted us to scrutinise MIC-A/B expression on uveal melanoma. To this end, we demonstrate stage-dependent expression of MIC-A/B in uveal melanoma.

## EXPERIMENTAL PROCEDURES

### Tissue samples

Tumour specimens of primary and metastatic uveal melanoma were obtained by surgical excision. The representative parts of the lesions were used for histological diagnosis and the immediate, next slides were used for scientific workup. Informed consent was obtained from all patients prior to any of these measures. The clinical course of the patients is summarised in Table 1

**Table 1:** Clinical course of patients with metastatic lesions

**Identifier**	**Primary tumour and therapy**	**Metastases**	**Therapy**	**Death**
4330/99 2854/99	1/96 enucleation	6/98 liver metastases 1/99 lung metastases	Fotemustine treosulphan/gemcitabine	1/00
800/98	3/91 enucleation	1/96 spleen metastases 8/96 liver metastases	Splenectomie interferon fotemustine	Date last seen 10/97
8202/97	96	9/96 liver and lung metastases	Fotemustine, IL-2 dacarbacin	1/98
9057/97b	12/89 radiation	8/94 lung metastases 97 abdominal metastases 98 brain metastases	Combined IL-2 and interferon alpha polychemotherapy	98
9059/97	91	5/97 skin and liver metastases 98 bone metastases	Fotemustine IL-2 Interferon alpha	11/98
1709/94	87 operation	9/93 brain, lung, liver metastases	Radiatio fotemustine	1/95
5368/92	87	2/92 liver metastases 11/92 cutaneous metastases	Interferon alpha McClay	5/93
15/99-2	1/99 enucleation	6/00 liver and lung metastases	Treosulphan and gemcitabine 10/01 fotemustine	9/02

.

### Immunohistochemistry

In total, 5 *μ*m sections of paraffin embedded tumours were treated two times with xylol for 10 min at room temperature. Subsequently, sections were washed three times with ethanol followed by one rinse with distilled water. For antigen retrieval, sections were incubated with citrate buffer, pH 6.0 (DAKO, Hamburg, Germany) for 30 min at 90°C and then air dried for 20 min at room temperature. Next, slides were rinsed twice with phosphate-buffered saline (PBS, DAKO, S3024) and thereafter incubated with Blocking Solution (DAKO, S2023) for 10 min at room temperature. After two additional washing steps with PBS for 10 min at room temperature each, the respective primary monoclonal antibodies (mAb) in PBS/1% bovine serum albumin (BSA) were added to the sections, which were then incubated for 30 min at room temperature. The following mAb were used: MIC-A/B (6D4, murine IgG1), NKG2D (5C6), CD57 (Klon NK-1, Zytomed, Berlin, Germany), CD8 (DAKO, Hamburg, Germany), CD3 (DAKO, Hamburg, Germany); the respective predetermined dilutions ranged from 1 : 50 to 1 : 800. After two washes with PBS for 10 min each, biotinylated species-specific secondary Ab (Dako) were added to the sections. After 25 min the slides were washed twice with PBS/BSA, and finally the bound antibodies were visualised using streptavidin-HRP or -AEC (DAKO) according to the manufacturer's guidelines.

Histological evaluation and scores were assessed by independent observers (CSV and JCB) to ensure accuracy of quantification of immunohistochemical slides. During a simultaneous session, two observers using the same scale subsequently counted the positive cells per perspective in high-power magnification. A total of 500 cells were examined in at least five areas and the percentage of positive cells was determined to one of the following categories: −: none, (+): single, +: >40%, ++: >80% positive cells (Lo *et al*, 2003).

The mAb used for MIC-A/B (6D4) and NKG2D (5C6) were kindly provided by Veronica Groh and Thomas Spies, Fred Hutchinson Cancer Research Center, Seattle, USA and its characteristics have been described (Bauer *et al*, 1999; Groh *et al*, 1999, 2003; Steinle *et al*, 2001).

## RESULTS AND DISCUSSION

**Figure 1:**
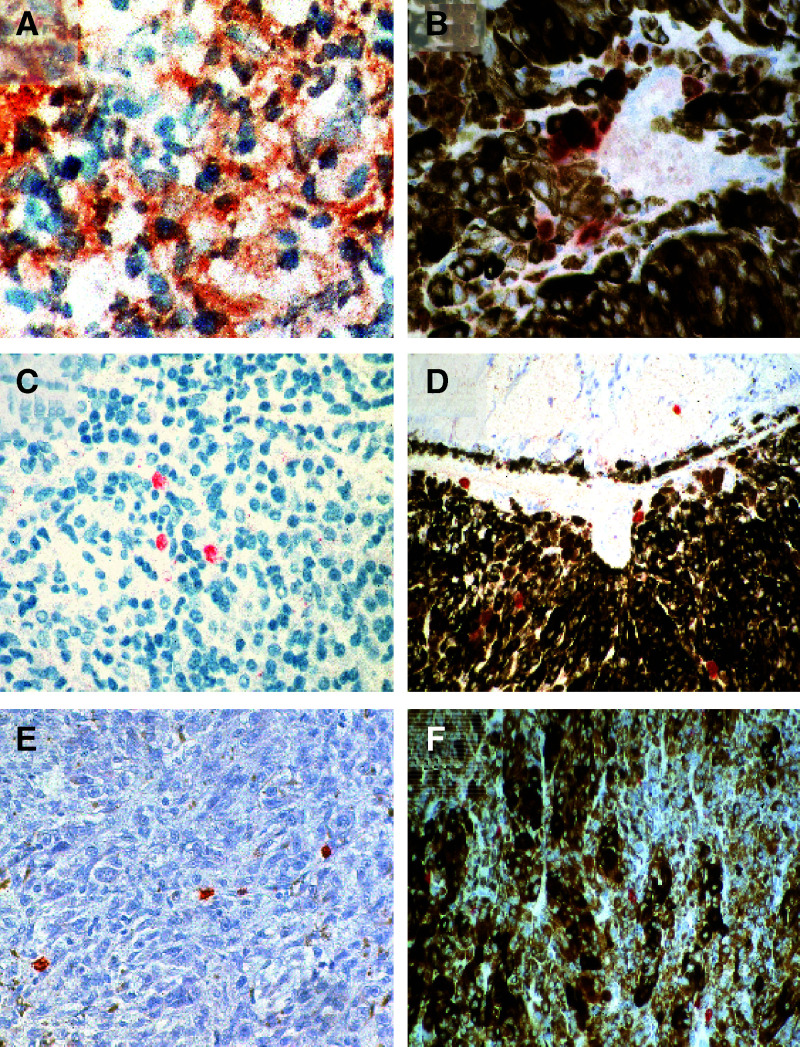
Expression of MIC (**A** and **B**), on two primary uveal melanomas in relation to the presence of infiltrating lymphocytes CD57 (**E** and **F**) and the expression of the activating killer receptor NKG2D (**C** and **D**).

Nine primary uveal melanomas and 11 metastases were analysed by immunohistochemistry for MIC-A/B and NKG2D expression (Figure 1

). It should be noted that we did not perform a longitudinal study analyzing primary tumours and consecutive metastatic lesions.

We detected MIC-A/B expression on 50% of the primary tumours (Table 2

**Table 2:** Uveal melanoma – primary tumours

		**MIC-A/B^b^**		**NKG 2-D^c^**		**CD 57^c^**	
**No.**	**TIL^a^**	**Frequency**	**Intensity**	**Frequency**	**Intensity**	**Frequency**	**Intensity**
401/99	Ø	−		−		−	
331/99	(+)	+^d^	+	+	(+)	(+)	+
133/99	(+)	(+)	(+)	(+)	+	(+)	+
15/99	+	−		−		−	
346/98	Ø	−		−		−	
401/97	(+)	−		−		−	
290/97	(+)	++	+	++	+	++	+
269/97	(+)	+	+	+	+	+	+
120/92	+	+	+	+	(+)	+	+

a The inflammatory infiltrate is classified according to the criteria of *Elder and Clark* as ++: brisk; +: nonbrisk; (+): dim; Ø: absent.

b Expression on melanoma cells.

c Expression on tumour infiltrating lymphocytes.

d −: none, (+): single, +: <40%, ++: <80% positive cells.

). The corresponding receptor NKG2D was expressed on a subset of tumour infiltrating lymphocytes present in all MIC^+^ tumours (Table 2). The NK cell marker CD57 was present on majority of infiltrating cells in the MIC/NKG2D positive primary tumours (Figure 1). Interestingly, the magnitude of the inflammatory infiltrate directly correlated with the expression of MIC-A/B on the tumour cells.

In contrast, none of the 10 cutaneous metastases from untreated patients expressed MIC-A/B (Table 3

**Table 3:** Uveal melanoma – metastases

		**MIC-A/B^b^**		**NKG 2-D^c^**		**CD57^c^**	**CD3^c^**
**No.**	**TIL^a^**	**Frequency**	**Intensity**	**Frequency**	**Intensity**	**Frequency**	**Frequency**
2616/99	(+)	–		–		–	+
2854/99^d^	(+)	–		–		–	(+)
4430/99	+	+^e^	++	++	+	+	++
800/98	(+)	–		–		+	+
8202/97	(+)	–		–		–	(+)
9057/97	(+)	–		–		+	++
9059/97	(+)	–		–		+	+
1709/94	++	–		–		–	–
3713/95	(+)	–		–		–	+
6830/95	(+)	–		–		(+)	+
7602/95	(+)	–		–		–	(+)
5368/92	+	–		–		(+)	–

a The inflammatory infiltrate is classified according to the criteria of *Elder and Clark* as ++: brisk; +: nonbrisk; (+): dim; Ø: absent.

b Expression on melanoma cells.

c Expression on tumour infiltrating lymphocytes.

d #2854/99 and 4430/99 were obtained from the same patient prior or after therapy with fotemustine.

e −: none, (+): single, +: <40%, ++: <80% positive cells.

). However, the magnitude of the inflammatory infiltrate in metastases was not correlated with the presence of MIC. This infiltrate largely consisted of CD3^+^ cells, but in 50% of the metastases a considerable number of CD57+ cells were present (Figure 2

**Figure 2:**
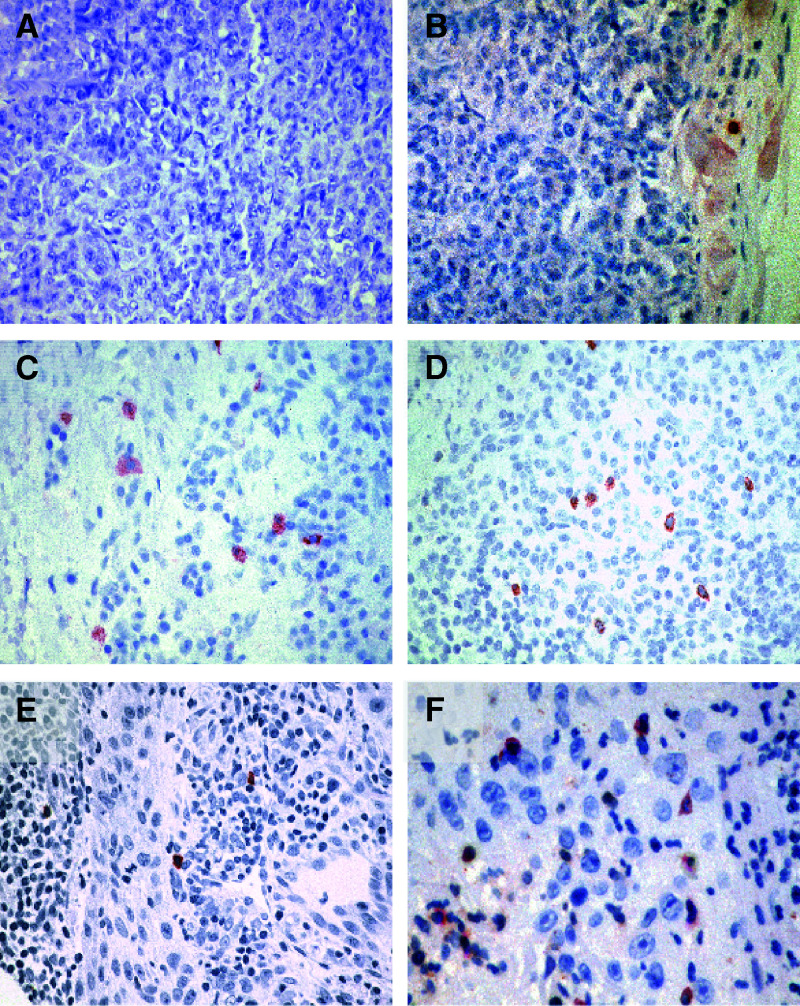
Expression of MIC (**A**, **C**), NKG2D (**B**, **D**), CD57 (**E**), and CD3 (**F**) on tumour or infiltrating cells of metastatic melanoma in relation to therapy with fotemustine. The sections **A** and **B** were obtained prior, and the sections **C–F** subsequent to therapy.

). Notably, none of these infiltrating cells expressed NKG2D. Due to the small number of cases we did not perform statistical analysis to correlate MIC-A/B and the magnitude of infiltrate, in order not to overestimate the power of our observation.

The quest to establish an effective therapy for metastatic melanoma still remains ajar. This notion holds true particularly for disseminated uveal melanoma, since improvements of the treatment of primary uveal melanoma, enabling the preservation of the eye and its visual function, has not reduced the rate of subsequent tumour dissemination. The range of therapeutic options for stage IV disease encompasses chemotherapeutic, immune, antiangiogenic, and hormonal strategies or a combination thereof. Although the rationale for such combinations, for example chemo-immune therapy, is mostly empiric chemo-immune therapy still is the most promising approach. Notably, the sequence in which chemo- and immune therapies are given, that is, first chemotherapy followed by immunotherapy or *vice versa*, strongly correlates with the therapeutic activity. The synergistic effect of this combination may be explained by the chemotherapy-induced expression of stress-induced molecules, for example MIC-A/B, on tumour cells, which serve as targets for immune stimulatory molecules such as NKG2D expressed on NK-, T- and NK/T-cells.

We were able to analyse two metastases from one patient, which had been obtained prior and subsequent to therapy with fotemustine, interferon *α* and IL2. Notably, prior to chemotherapy neither MIC-A/B nor its receptor NKG2D was detectable in the tumour lesion. After two courses of therapy, however, another subcutaneous metastasis from the same patient was characterised by an intense expression of MIC-A/B on more than 10% of the tumour cells. This metastasis was further characterised by a dense inflammatory infiltrate of CD3- and CD57-positive cells. This infiltrate also stained positive for NKG2D. Although it cannot be proved that these differences are merely due to lesion heterogeneity, the constant lack of MIC-A/B in all patients not receiving chemotherapy strongly suggests that MIC expression is indeed induced by the applied therapeutic measures.

The lack of correlation of melanoma-specific immune responses and the clinical course of the disease has provided the impetus to investigate the mechanisms used by malignant cells to escape immune recognition. In this respect, abnormalities in HLA class I expression have been conclusively demonstrated as one of these mechanisms (Campoli *et al*, 2002). Since NK cell lysis is enhanced by downregulated MHC class I expression, Uveal melanoma may actually represent an exception to this rule (Seliger *et al*, 2003). Indeed, Ma *et al* (1995) demonstrated that the sensitivity of human uveal melanoma cells to NK cell lysis was reciprocal to the level of HLA class I expression. Susceptibility to NK lysis also impacts the metastatic behaviour: injection of uveal melanoma cells into eyes of nude mice demonstrated an inverse correlation between development of hepatic metastases and the sensitivity of cell lysis *in vitro* (Ma and Niederkorn, 1995).

Since NK cells infringe in immune surveillance of uveal melanoma, immunotherapy for these tumours should include strategies influence them and therefore innate immunity. The interaction of NKG2D with MIC-A/B may serve this approach. The MIC gene transcriptional regulatory sequences contain heat-shock elements similar to those in the human Hsp70 promoter. Since genotoxic chemotherapeutic agents such as alkylating, cytotoxic, antimitotic and antimetabolic substances, activate the Hsp70 promoter (Hunt and Morimoto, 1985; Groh *et al*, 1996) via binding of HSF-1, thereby inducing gene transcription, similar mechanisms, may explain the induction of MIC-A/B expression by fotemustine (Liu *et al*, 1996). Though induction of MIC-A/B by chemotherapy and subsequent presence of an NKG2D^+^ inflammatory infiltrate was only investigated in one patient, our previous studies in cutaneous melanoma demonstrated changes in the inflammatory infiltrates at different tumour stages, which were associated with changes in MIC-A/B expression (Vetter *et al*, 2002), thus strengthening the relevance of these findings in this one patient.

In conclusion, MIC/NKG2D interactions are present in the initial phases of an immune response to uveal melanoma, but this interaction seems to be less involved in responses to metastatic disease suggesting immune selection of MIC negative tumour cells. However, cell stresses such as the presence of different cytotoxic agents seem to restore MIC-A/B expression. Even if this notion is based on a case report, it is of particular interest as it links chemo- and immunotherapy and explains the synergistic effects of these different therapeutic approaches empirically established in the clinic (Woll *et al*, 1999).
